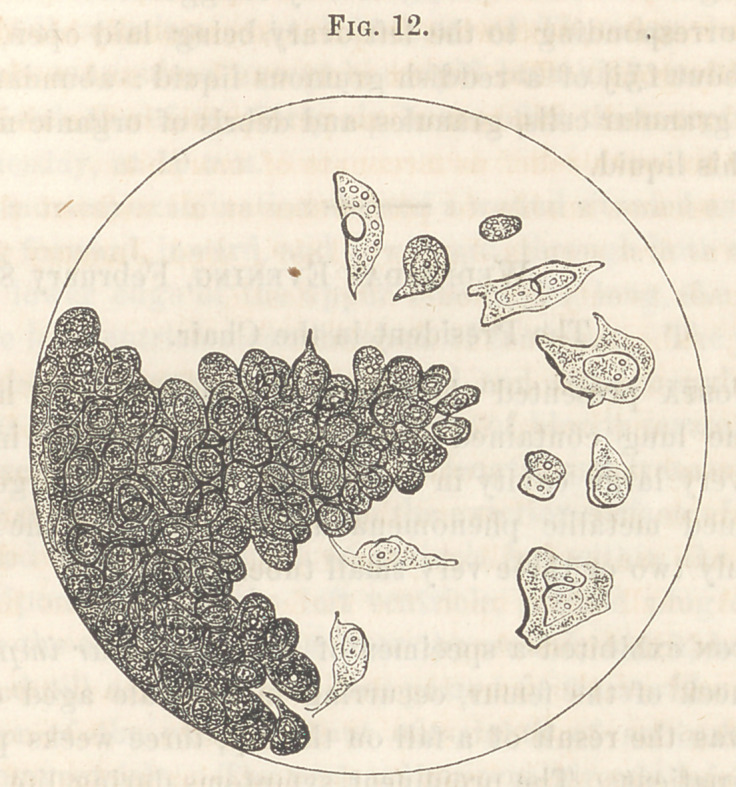# Proceedings of the Pathological Society of Philadelphia

**Published:** 1860-05

**Authors:** 


### ART. V.—*Proceedings of the Pathological Society of Philadelphia.*

WEDNESDAY EVENING, January 11th, 1860.

The President in the Chair.

*Fused Kidneys.*—DR. LENOX HODGE exhibited a large malformed kidney, consisting of two kidneys fused into one. It was taken from the body of a middle-aged man who died in the Pennsylvania Hospital, from traumatic meningitis.

The two kidneys were in reality perfectly normal, except that the pelves were directed more anteriorly than usual, and that their lower extremities were connected by a ribbon-like band passing across the vertebral column. There was no deviation from the usual position, as is so commonly the case.*

DR. DARRACH exhibited a tubercle the size of a marble, taken from the brain substance of a young girl who died of tubercular meningitis. Tubercles were found in the membranes of the brain and also in the lungs.

*Universal Melanosis.*—DR. GROSS, in exhibiting the specimens, said: The following case is one of unusual interest, from the almost universal diffusion of this morbid product. The number of similar instances upon record is very limited.

The patient, Elisha Chadick, aged fifty-eight, was a resident of Louisville; he was a stout, robust man, a pilot on the Ohio and Mississippi rivers, and first began to complain of indisposition in August, 1854. His attention was originally directed to the anus and rectum, in consequence of the existence of considerable pain and soreness, which induced him to believe that he was affected with internal hemorrhoids, an opinion somewhat strengthened by the fact that there was occasionally a slight discharge of blood. The symptoms steadily increasing, the general health at length gave way, and the man was obliged to take to his bed,

---

* See Dr. Morton's case, and Dr. Harris's remarks on it, in the Proceedings of this Society of May 12th, 1858.

which he never left until he died, a little upwards of a year from the commencement of his illness. The prominent symptoms, during the latter stages of his illness, were rapid emaciation and loss of strength, failure of appetite and sleep, an irritable state of the bladder, with a frequent desire to urinate, frequent discharges from the lower bowel of a mucous and bloody character, and considerable pulmonary and bronchial irritation, with occasional cough. The finger inserted into the rectum readily detected the presence of mechanical obstruction from some morbid growth, and bluish-looking tumors existed in the groin, and in the skin of different parts of the body.

The dissection was made by Professor T. G. Richardson and myself, nine hours after death. The body was excessively emaciated, and the hands, feet, and legs, were somewhat œdematous. The surface was studded with tubercles, upwards of fifty being counted on the abdomen, chest, and shoulders; a few also existed on the arms and legs, and one on the right temple. Ranging in size from a pea to a small hazel-nut, they were perfectly movable, of a flat, rounded, or spherical shape, and of a firm, gristly consistence. Most of them were situated in the subcutaneous cellular tissue, or in this tissue and in the substance of the skin. The largest, and apparently the oldest, were of a black color, the rest being whitish or grayish. The lymphatic ganglions of both groins were considerably enlarged, and of a black color; those on the right side being more deeply involved than the left. The axillary ganglions were similarly affected, only in a slighter degree.

The *omentum*, thickened and granulated, presented thousands of hard, black, shot-like tubercles, with here and there a whitish one, very vascular, and larger than the black.

The whole *peritoneal* surface, parietal as well as visceral, was studded with black tubercles, from the size of a small shot to that of an ordinary pea; very hard, flattened, and apparently developed in the subserous cellular substance, but projecting beyond the surrounding level. They were most abundant in the meso-rectum, on the ascending colon, and on the wall of the abdomen, opposite the liver. The diaphragmatic surface of the membrane, the stomach, and small intestines, were nearly free from disease. Upon laying open some of these tubercles, many of them were found to have a white central nucleus. No serous effusion existed in the peritoneal cavity.

The *liver* was of the normal size and weight, and, for the most part, of sound structure. A few small melanotic tubercles were seen upon its surface, just beneath its peritoneal investment. At the inferior border of the right lobe, by the side of the gall-bladder, were several black masses of a soft, pultaceous consistence, the largest being of the volume of a pullet's egg. Nearly a dozen small tumors, white and black, intermixed, and of scirrhous hardness, occupied the interior of the organ. The hepatic substance around these tumors did not appear to be materially affected.

The *gall-bladder* was somewhat larger than usual, and filled with viscid, greenish bile. Upon its inner surface were a number of very small tubercles, seated in the submucous cellular tissue, and of a black color.

The *spleen* was of the natural volume and consistence, but its interior contained a broad lamellar body, of a whitish hue, and of a fibro-cartilaginous consistence.

The *pancreas* was considerably hypertrophied, and contained a large number of black tubercles, from the volume of a small shot to that of a small hickory-nut.

The *stomach*, which was of the natural size, contained a number of tubercles, all of small volume, hard, and more white than black. One or two of them were partially ulcerated. The pyloric and œsophageal extremities of the organ were healthy, as was also the *œsophagus* itself.

The *bowel* was carefully examined in its entire length. A few small, black tubercles were found in the duodenum, and the upper portion of the jejunum. The ileum, ileo-cæcal valve and colon were sound, the latter containing healthy fæcal matter and some gas. The lower part of the rectum and anus were both much diseased, being of scirrhous hardness, melanotic, and ulcerated at the posterior surface. The anus was much contracted and very firm, the induration extending for a considerable distance around.

The *kidneys* were affected in different degrees. The left was one-third larger than natural, and rather distorted, numerous white and blackish tubercles being found both upon its surface and in its interior. The right organ was somewhat under the natural volume, and nearly sound, no cancerous deposits being discoverable. The ureters were healthy.

Both *supra-renal* capsules were deeply involved in the disease. The left was fully half as large as a sound adult kidney, very black, and much softened; the right was also much hypertrophied, black, and broken down.

The *urinary bladder*, containing about three ounces of turbid urine, was covered with small, black tubercles, lying in the subserous cellular and adipose tissues. Its inner surface was generally sound, but slightly fasciculated at the bas-fond, and presented five distinct, characteristic tubercles, the largest about the size of a filbert. The walls of the organ were one-third of an inch in thickness, very hard, nodulated on the surface, and filled with white and blackish tubercles, the two substances being much blended. The superior extremity was more free from disease than the rest of the organ.

The *prostate gland*, very hard, and somewhat enlarged, was composed of a mixture of white and black substance, the greater amount of discoloration being in front. The organ was two inches and a quarter in length, by two inches and an eighth in width, and one inch and an eighth in thickness.

The *seminal vesicles* had apparently undergone the fibrous degeneration, being very hard and dense, with hardly any remains of their natural cavity. They were free from melanotic deposits. The testicles were normal.

Finally, the *lymphatic ganglions* of the pelvis, especially those around the bladder, prostate gland, and rectum, were all in a state of enlargement, induration, and melanotic degeneration.

The *lungs* were deeply implicated in this remarkable disease. Black and white tubercles were numerously and extensively scattered over the outer surface of the right organ, which exhibited a linear, dark, mottled aspect, as is so often seen in health. The tubercles, which were situated in the subserous cellular substance, were hard and prominent, the largest being the size of a dime. The interior of the organ was crowded with similar bodies. The left lung, firmly and extensively attached to the ribs by ancient adhesions, contained tubercles similar to those in the right lung, but neither so large nor so numerous. Both costal *pleuræ* exhibited here and there, in their connecting tissues, small tubercles, both white and black. Their cavities, however, were free from serous effusion. The larynx, trachea, and bronchia were healthy.

The *bronchial* lymphatic ganglions were enlarged, indurated, and characteristically black.

The *heart*, which was of the normal bulk and weight, presented, both externally and internally, a number of black and white tubercles. The right auricle contained altogether about twenty of these bodies, some imbedded in its substance, others, and the largest, projecting from its surface. The smallest did not exceed the size of a mustard-seed. The greatest number were situated in the auricular appendage, in the pectiniform fibres. Most of them, especially the larger, were of a bluish color, a few only being white or grayish. Five similar bodies, some black and others white, were discovered in the right ventricle, among its fleshy columns, the largest being about the volume of a marrow-fat pea, and all very hard and dense. Five small, black tubercles also existed in the left ventricle, in and among the fleshy columns; and several were found in the left auricle, one externally and the other internally.

The *pericardium* was healthy, and no water was contained in its cavity.

The *aorta* was sound, except in its abdominal division, where there were distinct marks of earthy deposits and softening, with some degree of abnormal redness. The common iliac arteries were similarly affected, the left containing, in addition, a small melanotic spot, apparently situated in the subserous tissue. The vena cava presented nothing unusual.

The *thyroid gland*, considerably enlarged in bulk, and greatly indurated, contained a large amount of melanotic matter; the quantity being much greater on the right side than on the left, which was occupied with a great deal of white, crude substance.

The *brain* and spinal cord, eyes, bones, joints, and voluntary muscles were not examined, but there was no reason to believe, from anything that occurred during life, or during the progress of the dissection, that these several structures were at all implicated in the disease

The peculiarities presented by this case are, first, the extensive distribution of the heterologous matter, constituting thus a kind of carcinomatous diathesis; secondly, the combination of melanosis with scirrhus; thirdly, the gradual wearing out of the system without any marked diagnostic symptoms as to the true nature of the disease, until the appearance of the bluish tubercles in the skin and groins; and, lastly, the fact that although there was the most complete disorganization of the supra-renal capsules, yet there was no bronzed appearance of the surface.

*Fracture of the Spine.*—DR. BRINTON directed the attention of the Society to a series of specimens removed from the body of a man who had recently died at the St. Joseph's Hospital, of this city. Some five months previous, the patient, a man about twenty-five years of age, had been accidentally struck on the back by a revolving fly-wheel. The result of this injury had been an impacted fracture of three dorsal vertebræ, and a luxation of the body of the sternum from the manubrium. Dr. Brinton exhibited casts of the parts, taken during life, and alluded to the extreme rarity of luxations occurring between the sternal bones. The further consideration of this injury, with a full history of the case and an account of the reputed parallel cases, he stated he would at some future time lay before the Society.

A discussion took place between several members of the Society, as to the amount, if any, of reparative process set up in the fractured vertebræ. The subject was finally referred to a committee.

---

WEDNESDAY EVENING, January 25th, 1860.

The President in the Chair.

*Hypertrophy of the Prostate Gland.*—This specimen was presented by DR. LENOX HODGE. The man from whom the specimen was taken was admitted into the hospital, for an acute attack of retention of urine. He was sixty-eight years of age; and, when received, was already in a low typhoid condition, from which he did not rally. He died on the sixth day. The urine always contained a large deposit of mucus and pus. A catheter was frequently passed into the bladder. By bearing in mind the probable enlargement of the middle lobe of the prostate, and the position of what was thought to be a false passage, this was readily effected. Upon the post-mortem examination it was found that the whole of the prostate gland was hypertrophied, especially the middle lobe, through the centre of which there was a false passage almost entering the bladder. The urethra, in the prostatic portion, was much dilated, and passed in front of and thus over, and also to the left of the middle lobe of the prostate. The walls of the bladder were hypertrophied and the ureters dilated.

*Fibrous Tumor of the Left Labium.*—DR. GROSS exhibited a large tumor, weighing upwards of three pounds, removed from the left labium of a colored woman twenty-six years of age. On section, a clear fluid oozed out. The appearance of the mass was that of a yellow substance resembling fat, intersected with large white bands. The swelling had been forming for upwards of two years and a half. It was the size of a child's head at full term, and occupied the left labium descending from between the vagina and the tuberosity of the ischium to nearly the middle of the thigh. It felt like a smooth, firm, and elastic body. The tumor was completely extirpated while the patient was under the influence of chloroform.

DR. HODGE mentioned a similar case which he had seen in the hospital, in which the tumor was pyriform and attached by a pedicle to the left labium; it was even larger than the one exhibited by Dr. Gross. The diagnosis was a fibrous tumor; but the patient, a young married woman, refused to submit to an operation, and left the hospital.

*Cancer of the Heart.*—DR. GROSS presented a number of specimens of *encephaloid cancer* which had been taken after death from a child six years of age, a patient of Dr. C. Osler, of this city. The only prominent symptoms in this case had been ascites, emaciation, and a tumor at the right side of the neck; no physical signs of disease of the heart were noticed. Post-mortem section, however, discovered masses of encephaloid tumors growing from the peritoneum epiploon mesenteric glands, pancreas, kidneys, and left auriculo-ventricular orifice. The stomach, supra-renal capsules, pleura, and liver were healthy; there was no hereditary predisposition to cancer.

*Cancer of the Bladder.*—DR. PACKARD exhibited a specimen of this affection removed from a patient of Dr. Dunton:—

Mrs. Young, aged forty-seven, American, housekeeper, had been sick four years, and had been complaining more or less for a year past. She has had occasional flooding, and pains in her back, a constant boring sensation in the uterus, and occasionally lancinating pains. The os uteri was tender on pressure; for some time past, bloody discharge came on irregularly. The bowels were constipated; the passages painful; the urine was free, somewhat frequent, occasionally bloody; general health tolerably good. The cervix uteri was hard and nodulated; the anterior lip elongated. No tumor was perceptible in the abdomen. No anodyne gave so much relief as meconate of morphia.

She finally became unable to pass water at all without the catheter. The patient was first seen in January, 1859; died, January 22, 1860.

*Autopsy*, made twenty-six hours after death.

Body pale; subcutaneous fat abundant.

Upon opening the abdomen, the bladder was found largely distended with blood-clots, and bound firmly to the anterior surface of the uterus. Its mucous membrane was studded with spots of cancerous deposit, of extremely variable size. Toward the left margin of the vesical triangle there was a small mass of villous cancer projecting up into the cavity of the organ. A small ulcer upon the surface of this mass had perhaps been the seat of the hemorrhage.

The uterus and ovaries were blended into a firm mass, two cysts posteriorly marking the respective sites of the ovaries. Between these two cysts, and bound somewhat firmly to the posterior surface of the uterus, ran the rectum, forming a double curve in front of the sacrum. The os uteri was very indistinct, the vagina shortened and much firmer in texture than normal.

The rectum seemed perfectly healthy; the small intestine was adherent at one point to the fundus of the uterus.

The ureters were very much distended with limpid urine, as if obstructed below. I was unable to detect any passage from them into the bladder; their walls were thinned. The kidneys were atrophied, their calices and infundibula very largely distended, and their secreting structure greatly lessened in quantity. They were very pale—in fact, apparently almost bloodless.

The supra-renal capsules were the seat of a deposit, whitish, in distinct granules about as large as a pin's head, without any special microscopic characters.

The liver was healthy, but on its surface were half a dozen masses of cancerous deposit, of a clear white color, projecting somewhat. On the corresponding surface of the diaphragm were similar masses.

Pancreas, lungs, and spleen healthy.

*Microscopic Examination.*—All the cancerous deposits alluded to consisted of cells, generally ovoid, many of them pointed, of very various shapes and sizes, with large, nucleolated nuclei. Some of the cells contained two nuclei, some of the nuclei two or more nucleoli. Some of the cells were in a state of degeneration, having lost their contents, or at least having faded very much, and showing here and there a few small oil-drops.

FIG. 12.

The fungous growth at the base of the bladder was seen, on letting the specimen lie in water, to be flocculent like the outer surface of the chorion. On examining these flocculi, they were found to consist of vessels with enormous numbers of cancer-cells, meshes of wavy and very fine fibres, and with projections very like the intestinal villi along their margins. These latter projections seemed to have a central stem, probably a loop of a capillary, around which were grouped the cells, generally attached by their smaller extremities, and strongly resembling tacks or iron-filings around the end of a magnet.

Such an arrangement as that just detailed is not mentioned, so far as I know, in any work on pathology. Wedl speaks of villous cancer, but describes the cells as lying parallel to the axes of the papillæ. Dr. Wilks, in his lectures on pathological anatomy, delivered at Guy's Hospital, London, and published in 1859, says that the villous character of a growth is secondary, and may be implanted either on an innocent or a cancerous tumor. He then goes on to describe a case of death from hematuria, in which what appeared like little tufts of moss were found growing from the mucous membrane of the bladder. When examined by the microscope, these "presented villous processes, and, what is very striking, as you may see in this drawing, which I made at the time, the surface is covered by columnar epithelium, long, battledore-shaped nucleated cells, very different from the ordinary epithelium of the bladder. Each villus, I should have said, contained loops of blood-vessels."

The drawing I have shown is in no way exaggerated.

The cyst corresponding to the left ovary being laid open, was found to contain about f℥ij of a reddish grumous liquid: abundant plates of cholesterine, granular cells, granules, and débris of organic matter, were detected in this liquid.

---

WEDNESDAY EVENING, February 8th, 1860.

The President in the Chair.

DR. DA COSTA presented the lungs of a woman who had died of phthisis. One lung contained many scattered tubercles in the lower lobe, and a very large cavity in the upper, sufficiently large indeed to have occasioned metallic phenomena during life. In the other lung there were only two or three very small tubercles.

DR. BRINTON exhibited a specimen of *extra-capsular impacted fracture* of the neck of the femur, occurring in a female aged eighty-four. This injury was the result of a fall on the hip, three weeks prior to the death of the patient. The prominent symptoms during life were shortening of the limb, eversion of the foot, and extreme rigidity. An examination of the specimen exhibited an irregular line of fracture extending obliquely across the bone, and splintering the great trochanter. The fractured extremity of the cervix was firmly impacted within the upper extremity of the shaft. No reparative action seemed to have been set up. Dr. Brinton made some remarks on the difficulty of diagnosis often met with in the occurrence of impacted fracture of this bone.

*Wound of Left Ventricle of the Heart; Death thirty-six hours after Injury.*—DR. LENOX HODGE said: On Wednesday afternoon, February first, H. W. was brought to the hospital, with a penetrating wound of the chest. It was said that he had been stabbed the evening before, and at the time had lost a large quantity of blood. At his admission he was cold, the pulse small and feeble, the respiration rapid, and his countenance expressive of great anxiety. The wound was situated between the fifth and sixth ribs, and a little to the left of the left nipple. It was an inch and a half in length; it penetrated forward, inward, and downward. Air and blood passed freely from and into the pleural cavity during respiration. There was no cough nor spitting of blood. The vesicular murmur could be heard, but the sound on percussion over the whole of the left side of chest was dull, the action of the heart was feeble and irritable. The mind was perfectly clear. During the afternoon and evening the skin became warmer, but the other symptoms remained unchanged. About midnight, the pulse was still feeble, and he was unable to speak; then he became exceedingly restless, and continued to roll incessantly till morning. At 7.30 A.M. of Thursday, he was almost pulseless and motionless, except his rapid breathing, and he died at 10 A.M. He lived, therefore, thirty-six hours after the reception of his injury, on Tuesday, at 10 P.M.

The post-mortem examination showed a wound situated as above stated, and passing forward, inward, and downward, through both surfaces of the pleura, the lower edge of the upper lobe of the lung, the pericardium, and into the left ventricle near the apex of the heart. The pleural cavity contained about ten ounces of fluid blood, and some coagula. The pericardium was filled with about three ounces of bloody serum. The wound of the left ventricle was half an inch in length, and transverse to the external layer of muscular fibres. On the exterior surface of the heart the wound gaped about one-fourth of an inch, but within the sides were in contact. Upon opening the left ventricle, a little plug of lymph was seen among the columnæ carneæ over the wound, which, however, could not be found till a blunt probe was passed in from without. Only the lower corner of the wound, about one-eighth of an inch, entered the cavity of the ventricle. The pericardium was already injected and covered with minute elevations.

*Softening of the Fornix and Septum Lucidum; Fatty Degeneration of the Heart.*—DR. KEATING said: I was called to attend Mr. H. D. B., on the twenty-eighth of December last. He was thirty-seven years old, and naturally of a strong constitution; had suffered on previous occasions from attacks of acute rheumatism, and was subject more or less to flying rheumatic pains, for which I had prescribed for him some weeks previous to his last illness.

Mr. B.'s health had been recently much impaired from mental distress consequent upon unsuccessful commercial operations, and he had been affected with general debility which went on daily increasing. I had seen him on several occasions at my office, and prescribed for a hacking cough which had annoyed him considerably, and impaired his strength by the violence of the paroxysms, causing him constantly to void the contents of his stomach.

On the morning of the twenty-eighth, I found him suffering from general rheumatic pains, tongue coated, abdomen free from pain, and no fever During the first two or three days he scarcely appeared ill, but was plunged into a profound melancholy.

On Saturday, the thirty-first, he seemed much relieved; the bowels had been freely opened, and although very much dejected, he was somewhat less gloomy than on previous visits.

On Monday, the second of January, he complained of a violent pain in the head, confined to the right frontal region, periodic in its character, and so intense as to cause him to groan out with the agony he endured, and to entreat for remedies which would cause him to sleep. This frontal and facial pain, which seemed decidedly neuralgic in its nature, playing around the branches of the nerves of the forehead and face, originated at one time in the ear and in the eye of the right side, and always commenced about 7 P.M., and lasted till 4 A.M.; then came an interval of four or five hours with positive relief from suffering, but followed by excessive prostration. This periodical headache now seemed to be the prominent symptom in his case. For several days it baffled all attempts made to relieve it, and he was becoming rapidly exhausted from want of sleep and from the violence of the pain.

On Wednesday, the fourth instant, Dr. La Roche saw him with me; the intense headache was then somewhat relieved, but he complained of a severe pain down the spine, especially in the cervical region, and he described the pain at times as resembling electrical shocks. He was very restless; at one time getting up, anon lying down, and complaining of any sudden motion as aggravating the pain in the head, which was very much like an iron bar pressing upon his brain; tongue natural; no fever; pupils natural; respiration rather more frequent; action of heart more rapid, but weak, with a decided blowing sound during the second sound; secretions natural; great disinclination to talk; a general tremulousness of the muscles, and vision slightly impaired.

Thursday, 5th.—The blister on the back of the neck dressed with morphia, relieved all the spinal and frontal pain, and caused him to pass a comfortable night. Pupils more dilated, and do not respond to the light; countenance pale, sunken, and haggard; hands exceedingly tremulous; staggers in walking; complains of double vision; secretions natural; tongue slightly furred; pulse more frequent, rather weak, but no fever, and seems to depend upon excessive irritability of the heart. He seems very much dejected and apprehensive, and very feeble, the least movement increasing perceptibly the respiration and circulation. He still continues to rise every day and dress himself, and his appetite so far has been very good, seeming to enjoy all the food which is allowed him. During the evening of this day he was suddenly attacked with great restlessness and anxiety; his condition was so alarming as to cause his wife to send for Dr. Gerhard, who lived in his immediate vicinity. Dr. Gerhard, considering the attack as hysterical, prescribed an anodyne, which was attended with a happy result.

Friday, 6th.—Passed a sleepless night; does not complain of any particular pain, but seems much more depressed; speech is rather thick; forgets words; tremulousness of muscles of the whole body; is exceedingly restless, and has great difficulty in moving about, although he is dressed and lies upon a lounge; tongue almost natural; pulse frequent, but not tense; breathing rapid; heart's action quick, but no force; face pale and haggard; conjunctivæ not the least injected; pupils dilated; double vision increased; has slight alienations of intellect, seeing strange figures near him, and discoursing on various subjects; bowels open by an enema; urine rather scanty, and contained a slight trace of albumen.

Saturday, 7th.—Tongue moist, slightly furred; pulse frequent; no fever; skin moist; spent a very restless night, constantly getting out of bed; pupils dilated; mind confused; some subsultus; double vision continues; complains that he is losing his mind; forgets words, and seems to have some difficulty in speaking; no paralysis of any portion of the body; fears that he is idiotic; has no particular pain; respiration frequent; his debility has increased; craves food, and takes it with pleasure.

9th, A.M.—This day he gradually grew worse; sleeplessness increased; incessant jactitation; double vision; pupils dilated; mind more and more confused; speech difficult; pulse frequent, but no heat of skin; face more haggard, with slight fever on the brow; tongue good; heart beats rapidly, but without force, and with distinct blowing sound; secretions generally natural; no costiveness; no paralysis; complains most of want of sleep, and excessive prostration; says the pain in the head is relieved; craves food, and enjoys it; continues to get up every day, dress himself, and to rest himself on the lounge in the back room; not much inclined to talk; remains in a partially comatose condition, but is easily roused, and, save for the loss of words and difficulty in speaking, seems quite intelligent.

His condition grew gradually worse, and he seemed to sink until Thursday, the twelfth, when his skin became hot and dry, and he had considerable fever; face flushed; pupils continue dilated; left eye a little more prominent; constant twitching of muscles; more comatose, and at times delirious; no paralysis; tongue more furred, and slightly red at edges; secretions almost natural. He now grew rapidly worse, and on Saturday, the fourteenth, presented all the symptoms of effusion having taken place on the brain; he became more comatose; great subsultus tendinum, threatening convulsions; could be roused, however, and gave some signs of intelligence until Monday night, up to which period he always arose to void his bowels, and always indicated his desire to pass water. He died on Tuesday, the 17th of January, at 3 P.M.

*Autopsy*, made by Dr. Packard, twenty-six hours after death.

The body was very muscular, and the subcutaneous fat abundant. The genital organs were extremely small.

On opening the *head*, the dura mater was found somewhat adherent to the skull-cap. In the subarachnoid space, at the upper surface of both cerebral hemispheres, and at the outer surface of the left, were deposits of yellow lymph. The fissure of Sylvius, on each side, was obliterated by adhesions. Between the optic chiasm and the pituitary body, in front of the infundibulum, was a mass of tough lymph about the size of a large pea; the pituitary body was flattened down by its pressure.

The brain substance was somewhat congested; the anterior lobe of the left hemisphere was softened; the corpus callosum, fornix, and septum lucidum were softened to a very marked degree, the latter being partly broken down. Nothing abnormal was observed in the ventricles.

In front of the upper part of the cerebellum, just below the velum interpositum, and filling up the third ventricle to a considerable extent, was a mass of quite a firm substance, slightly lobulated, and crying under the knife. An artery which ran through this mass was found, upon microscopical examination, to be perfectly healthy.

The deposits now mentioned, being placed under the microscope, were found to be composed of lymph in various stages of organization, from mere fibrilation to completely-formed fibrous tissue.

The *thorax* was next examined. Ossification had taken place in the costal cartilages. Both lungs were studded throughout with miliary tubercles; the right pleura was universally adherent. On the left side the fissure between the upper and lower lobes was complete, dividing the organ into two distinct portions. The heart was loaded with fat, and its muscular substance had undergone very marked fatty degeneration

In the abdomen, the liver only was examined; it was somewhat fatty.

DR. HEWSON exhibited a fibrous tumor of the uterus, and tubercular ulcerations of the intestine, removed from the same patient.

DR. KEATING drew attention to the fact that, in cases of fibrous tumors, an icteroid tract was frequenly present, which caused the case to be mistaken for cancer.

DR. LENNOX HODGE stated that a similar observation had been made by Professor Hodge.

## Figures and Tables

**Fig. 12. f1:**